# RhoA, Claudin 18, and c-MET in Gastric Cancer: Clinicopathological Characteristics and Prognostic Significance in Curative Resected Patients

**DOI:** 10.3390/medsci10010004

**Published:** 2021-12-29

**Authors:** Marina Alessandra Pereira, Marcus Fernando Kodama Pertille Ramos, Andre Roncon Dias, Leonardo Cardili, Renan Ribeiro e Ribeiro, Tiago Biachi de Castria, Bruno Zilberstein, Sergio Carlos Nahas, Ulysses Ribeiro, Evandro Sobroza de Mello

**Affiliations:** Instituto do Cancer, Hospital das Clinicas HCFMUSP, Faculdade de Medicina, Universidade de Sao Paulo, Sao Paulo 01246-000, Brazil; marcus.kodama@hc.fm.usp.br (M.F.K.P.R.); andre.dias@hc.fm.usp.br (A.R.D.); leonardo.cardili@hc.fm.usp.br (L.C.); r.ribeiro@hc.fm.usp.br (R.R.e.R.); tiagobiachi@yahoo.com.br (T.B.d.C.); bruno.zilberstein@hc.fm.usp.br (B.Z.); sergio.nahas@hc.fm.usp.br (S.C.N.); ulysses.ribeiro@hc.fm.usp.br (U.R.J.); evandro.mello@hc.fm.usp.br (E.S.d.M.)

**Keywords:** gastric cancer, claudin 18, Ras Homolog Family Member A, c-Mesenchymal–Epithelial Transition, immunohistochemistry

## Abstract

**Background:** Recently, markers related to molecular classification were suggested as promising therapeutic targets for treatment and prediction of prognosis in gastric cancer (GC), including c-MET, RhoA, and Claudin-18 (CLDN18). This study aimed to investigate their expression in GC and its correlation with clinicopathological characteristics and survival. **Methods:** We retrospectively evaluated GC patients who underwent curative gastrectomy. c-MET, RhoA, and CLDN18 were analyzed through immunohistochemistry (IHC), and groups for analysis were determined according to the median values obtained for each marker. **Results:** Among the 349 GC evaluated, 180 (51.6%), 59 (16.9%), and 61 (17.5%) patients were completely negative for c-MET, RhoA, and CLDN18, respectively. Total gastrectomy, D1 lymphadenectomy, poorly differentiated histology, and greater inflammatory infiltrate were more frequent in the c-MET-negative group. Diffuse type, greater inflammatory infiltrate, and advanced pT and pTNM stage were associated with low-RhoA GC. The venous invasion was more frequent in the low-CLDN18 group. Furthermore, c-MET was positively correlated with RhoA and negatively with CLDN18. HER2 expression was associated with c-MET-positive and high-CLDN18 GC; and loss of E-cadherin expression in c-MET-negative and low-RhoA GC. c-MET-negative and Low-RhoA were significantly associated with worse disease-free survival. **Conclusions:** c-MET, RhoA, and CLD18 expression occurred frequently in GC. RhoA GC had distinct clinicopathological characteristics related to prognosis. c-MET and RhoA were associated with survival but were not independent predictors of prognosis.

## 1. Introduction

Gastric cancer (GC) is one of the most common malignancies and the third leading cause of cancer death worldwide [1]. Surgical resection and lymphadenectomy remain the mainstay of treatment for curing, and the tumor stage provides important prognostic prediction [2,3].

Recently, advances in the knowledge of GC biology next to the integrative genomic analysis led to the proposal of molecular classification of GC into four subtypes, which have implications for patient treatment and prognosis. Among the subtypes, Microsatellite-instable (MSI) and Epstein–Barr virus (EBV)-positive GC are the most discussed and characterized. Both have recognized predictive biomarkers, including PD-L1 expression, and have been important in the field of target therapies such as immune checkpoint inhibitors [4,5,6,7]. MSI was even included as an additional prognostic factor in the 8th edition of TNM [8]. 

Conversely, the GCs defined as genomically stable (GS) and chromosomal-instable (CIN) represent more heterogeneous subtypes, bringing great challenges to the successful treatment of this disease. Accordingly, it shows that GC still requires a better knowledge of its molecular characteristics to identify new specific prognostic biomarkers based on the gene expression profile [4,9].

The TCGA study reported that CIN represents most of GC, and usually shows TP53 mutated and amplification of genes encoding receptor tyrosine kinases (RTK), as HER2. In addition, the c-Mesenchymal–Epithelial Transition (c-MET)—a proto-oncogene that encodes the Hepatocyte Growth Factor (HGF) receptor—stands out as a potential option for investigation [4,10]. Further, in the GS subtype, the TCGA study showed that diffuse histology, mutations in the E-cadherin gene (CDH1), mutations in the Ras Homolog Family Member A (RhoA) gene—a GTPase family involved in several biological processes; and fusions involving Claudin-18 (CLDN18)—a component of tight junctions; were enriched in this subtype of GC [4,10]. 

Besides assisting in the characterization of GC subtypes, these markers also play a role in tumor development and progression, and the relationship of their expression with the patients’ prognosis has also been the target of investigations [11,12,13,14]. Furthermore, targeted therapies for GC, such as targeting c-MET, RhoA, and CLDN18 are currently under investigation [9,11,12,13,15]. However, data on these markers in GC are still scarce, so knowing their characteristics is crucial to improving the therapeutic effectiveness and survival of these patients.

Thus, we investigated the expression of the tumor markers c-MET, RhoA, and CLDN18 by immunohistochemistry (IHC) in a large and extensively characterized Western cohort of resected GC and its correlation with clinicopathological characteristics and survival outcomes.

## 2. Materials and Methods

### 2.1. Patients

Patients with GC who underwent gastrectomy at our Institution between 2009 and 2019 were retrospectively evaluated from our prospective maintained database. Patients that meet the following eligibility criteria were selected: (1) curative intent gastrectomy, (2) histologically confirmed gastric adenocarcinoma, and (3) formalin-fixed paraffin-embedded (FFPE) tumor samples available for analysis. Emergency surgery and unresectable tumors were excluded from the study.

Clinicopathological data including age, sex, preoperative blood test, body mass index (BMI), ASA classification, comorbidities by Charlson comorbidity index (CCI) [16], type of gastrectomy, tumor size (cm), Lauren’s type, degree of tumor differentiation [17], lymphatic, venous and perineural invasion, and the number of lymph nodes were retrieved. Pathological TNM stage was evaluated following the 8th American Joint Committee on Cancer/International Union Against Cancer (AJCC/UICC) staging system [8]. 

Surgical resection and lymphadenectomy were performed based on the guidelines of the Japanese Gastric Cancer Association (JGCA) and the national consensus on GC [2,3]. Postoperative complications (POC) were graded based on Clavien–Dindo classification [18], and values > 2 were considered as major POC. Adjuvant chemoradiotherapy (CRT) and adjuvant or perioperative platinum-based chemotherapy (CMT) were administered according to clinical indication (T3/T4 and/or N+).

Follow-up appointments were performed every 3 months for the first year, and every 6 months in the following years. The postoperative follow-up was carried out by regular outpatient visits, and exams for relapse detection were performed based on the presence of symptoms.

This study was approved by our Hospital Ethics Committee and was registered online at Plataforma Brasil (CAAE: 37009120.0.0000.0068). 

### 2.2. Tissue Microarray Construction (TMA) 

Hematoxylin and eosin (HE)-stained slides were reviewed by a pathologist to select representative tumor areas and paraffin-embedded tumor specimens were used to construct a tissue microarray (TMA). Core tissue samples (2 mm in diameter) were taken from individual FFPE gastric carcinomas (donor blocks) and arranged in a new recipient paraffin block (tissue array block) using a precision mechanized system (Beecher Instruments, Silver Springs, MD, USA). Three to six tumor cores and two non-tumor mucosa cores were sampled from each case. TMA blocks were cut into 4 μm sections and submitted to HE and immunohistochemical (IHC) staining. 

### 2.3. Immunohistochemistry

IHC was performed using the following primary polyclonal antibodies: anti-Claudin 18, anti-RhoA, and anti-c-MET. Immunoexpression of HER2 (clone 4B5) and E-cadherin (clone 36B5) were also examined.

Briefly, the TMA sections were dewaxed, deparaffinized in xylene, and rehydrated in graded alcohols. Subsequently, sections were submitted to heat-induced antigen retrieval using citrate buffer and endogenous peroxidase was blocked with hydrogen peroxidase (3%). Slides were incubated overnight at 4 °C with the primary antibody. Avidin-biotin-free short polymer-based peroxidase amplification system was used and diaminobenzidine was applied for the development of reaction products. All slides were counterstained with hematoxylin. 

Tumors were evaluated for c-MET according to histoscore (H-score) systems [14,19]. C-MET was defined based on the percentage of staining tumor cells, multiplied by the staining intensity (0–3). The intensity of the staining was classified as 0 (no membrane or cytoplasmic reactivity), 1+ (weak membrane or cytoplasmic reactivity), 2+ (moderate membrane or cytoplasmic reactivity), and 3+ (strong membrane or cytoplasmic reactivity). The maximum score was 300.

For RhoA and CLDN18, samples were defined as RhoA or CLDN18 positive if they showed specific staining with at least 1+ intensity in any fraction of tumor cells (‘any positivity’) [20]. The immunoreactivity of RhoA (cytoplasmic staining) and CLDN18 (membrane staining) was assessed according to the percentage of stained tumor cells (0 to 100%), regardless of intensity [21]. 

High and low-level groups were established based on the median value obtained with the IHC assessment as the cut-off. The median percentage of stained tumor cells for both RhoA and CLDN18 was 33%, defining the high- and low-expression groups. As the median H-score for c-MET was equal to 0, the groups were divided into c-MET-negative and c-MET-positive.

The intensity of the membrane staining for HER2 was evaluated according to the HER2 scoring system for GC as reported previously [22]. The HER2-positive group was determined as IHC3+ and IHC2+. IHC0 and IHC1+ were defined as HER2-negative cases [23]. 

E-cadherin was evaluated using a 3-point score. Scores 2 (cytoplasmic and membrane labeling) and 3 (membrane labeling) were considered as normal expression; scores 0 (complete loss) and 1 (cytoplasmic expression) were considered as loss of expression [24].

All tumor spots of each case were semiquantitatively evaluated, and the final result was defined according to the average of the spots. To investigate the association between the expressions of markers with patient’s characteristics, the cases were qualified into two groups according to the median values for each marker (% of tumor or H-score) and determined as low and high levels, as previously described.

Microscopic analysis was carried out by a conventional light microscope by two pathologists in a blinded manner. A third one was consulted in case of disagreement and, and the slides were reanalyzed by all investigators using a multi-headed microscope.

### 2.4. Statistical Analysis

Statistical analyses were performed by Statistical Package of Social Sciences 20.0 software (SPSS, Inc., Chicago, IL, USA). Categorical and continuous data were evaluated using the chi-square test and *t*-test or Mann–Whitney U test, respectively. Correlation coefficients (r) between protein expressions for c-MET, RhoA, and CLDN18 were estimated using the Pearson correlation method. Survival was analyzed using the Kaplan–Meier method and the curves were compared using the log-rank test. Disease-free survival (DFS) was calculated from the date of surgery until the date of recurrence. Overall survival (OS) was calculated from the date of surgery until the date of death of any cause. Patients alive were censored at the date of the last follow-up. Univariate and multivariate analyses by Cox proportional hazards method were performed to establish the risk factors for survival. All *p*-values were two-sided, and *p* < 0.05 was considered statistically significant. 

## 3. Results

A total of 349 GC patients met our inclusion criteria and were enrolled in the study. The mean age was 63.7 years, ranging between 25.7 and 87.1 years. Male sex accounted for 59.9% of cases. D2 lymphadenectomy was performed on 292 patients (83.7%) and 51.9% of patients underwent subtotal gastrectomy. Lauren’s intestinal and diffuse-type accounted for 51.3% and 48.7% of cases. The mean number of retrieved lymph nodes was 39.1 (SD ± 18.5), and most patients were stage as pTNM III (43.0%). Some form of CMT treatment was adopted in 53.3% of patients (12.9% and 47.3% received perioperative CMT and adjuvant CMT/CRT, respectively).

**Figure 1:**
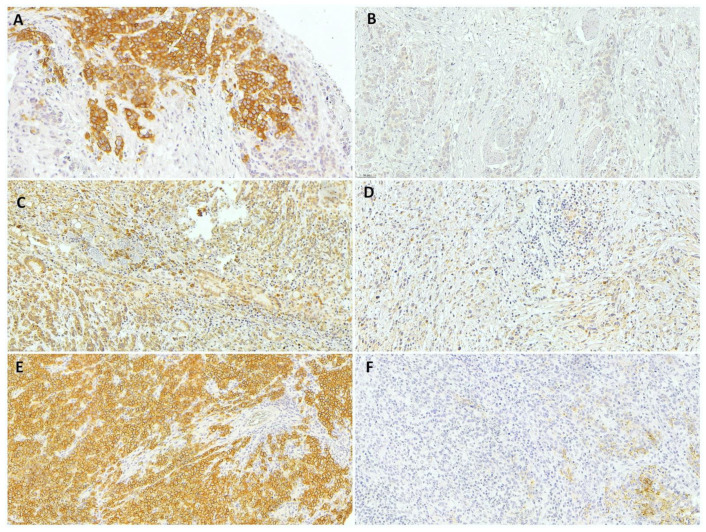
Representative results from immunohistochemical analysis of gastric adenocarcinoma. (**A**) c-MET-positive, (**B**) c-MET-negative, (**C**) high-RhoA, (**D**) low-RhoA, (**E**) high-CLDN18, and (**F**) low-CLDN18 gastric cancer (20× magnification).

According to the IHC results (Figure 1), 180 (51.6%), 59 (16.9%), and 61 (17.5%) patients were completely negative for c-MET, RhoA, and CLDN18, respectively. The mean percentage of positive tumor cells for c-MET, RhoA, and CLDN18 expression was 16.2% (SD ± 24, mean H-score = 20.7 ± 36.8), 36.2% (SD ± 30), and 35.0% (SD ± 29.9), respectively. Accordingly, the expression groups were established based on the median value obtained with the IHC assessment (median of 0 for c-MET and 33 for RhoA and CLDN18), as previously described in the methods sections (based on H-score for c-MET according to the percentage of stained tumor cells for RhoA and CLDN18).

### 3.1. C-MET and Clinicopathological Characteristics 

Among the 349 GCs evaluated, 180 (51.6%) were classified as c-MET-negative and 169 (48.4%) as c-MET-positive based on H-score. Clinicopathological characteristics are presented in Table 1. Total gastrectomy, D1 lymphadenectomy, poorly differentiated histology, moderate/intense peritumoral inflammatory infiltrate were more frequent in the c-MET-negative group compared to c-MET-positive patients. Lymph node metastasis and pT3/T4 GC was more common in the c-MET-negative group, but without reached statistical significance.

### 3.2. RhoA and Clinicopathological Characteristics

A total of 174 (49.9%) GCs were classified as low-RhoA and 175 (50.1%) as high-RhoA. Diffuse Lauren type, poorly differentiated tumors, moderate/intense inflammatory infiltrate, depth of tumor invasion, and advanced pTNM stage were associated with the low-RhoA group. Clinical and pathological characteristics of both groups are summarized in Table 2.

### 3.3. CLDN18 and Clinicopathological Characteristics

GC was defined as low-CLDN18 in 173 (49.6%) patients and as high-CLDN18 in 176 (50.4%) cases. Clinicopathological characteristics of GC according to CLDN18 groups are present in Table 3.

The presence of venous invasion was associated with the low-CLDN18 group. Depth of tumor invasion was also observed in low-CLDN18 patients, although without reaching statistical significance.

### 3.4. Immunohistochemical Analysis and Correlation between c-MET, RhoA, and CLDN18

**Figure 2:**
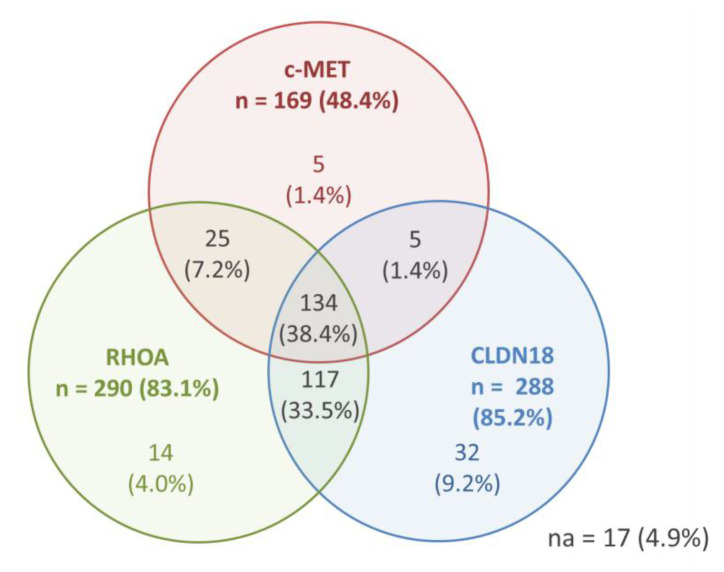
IHC results for c-MET, RhoA, and CLDN18—any positive expression and co-expression (*n* = 349 patients).

Results for the positivity of c-MET, RhoA, and CLDN18 (any positivity)—as well as the co-expression of the markers—are shown in Figure 2. Only 17 (4.9%) cases were completely negative for the three evaluated profile.

Considering the groups established for analysis (based on the mean values), tumors with c-MET-negative, low-Rhoa, and low-CLDN18 simultaneously was seen in 59 cases, while positive status for c-MET with high-RhoA and high-CLDN18 was observed in 52 patients. 

c-MET-positive GC was associated to high-RhoA (*p* < 0.001). No association was observed between c-MET and CLDN18 groups (*p* = 0.112), and between RhoA and CLDN18 (*p* = 0.422).

Furthermore, the Pearson correlation test demonstrated that c-MET was positively correlated with RhoA expression (r = 0.250 and *p* < 0.001), and negatively correlated with CLDN18 expression (r = −0.139 and *p* = 0.010). No correlation was observed between RhoA and CLDN18 expression (r = 0.023 and *p* = 0.663). (Supplementary Table S2)

Regarding the other markers analyzed in the study, HER2 hyperexpression was associated with c-MET positivity (*p* = 0.014) and high-CLDN18 (*p* = 0.046) GC. A higher frequency of loss of E-cadherin expression was observed in cases with c-MET-negative (*p* = 0.013) and low-RhoA (*p* = 0.040).

Pearson correlation test showed that HER2 was significantly correlated with c-MET expression (r = 0.168 and *p* = 0.002). No correlation was observed between HER2 expression and RhoA (r = 0.049 and *p* = 0.376) and between HER2 and CLDN18 (r = −0.107 and *p* = 0.052). (Supplementary Table S2) 

Considering the E-cadherin, significantly correlation was found between E-cadherin expression and c-MET(r = −0.122 and *p* = 0.028), and between E-cadherin and RhoA expression (r = −0.125 and *p* = 0.022). While there was no correlation between E-cadherin and CLDN18 (r = 0.025 and *p* = 0.652) (Supplementary Table S2).

### 3.5. Survival Analysis

**Figure 3:**
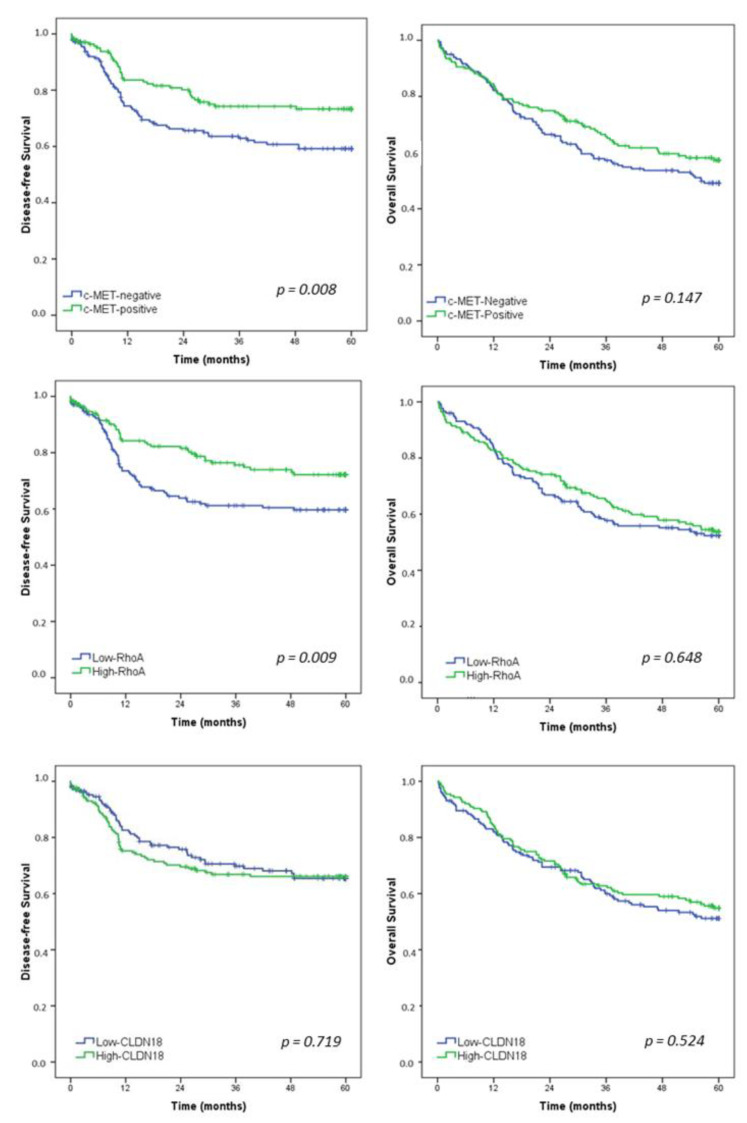
Kaplan–Meier survival analysis. Disease-free survival and overall survival in gastric cancer patients according to c-MET, RhoA, and CLDN18—Low vs. High groups.

After a median follow-up of 47.1 months, 105 patients had recurrence and 157 died. Estimated DFS and OS rates for the entire study population were 65.8% and 53.4%, respectively. Survival curves for all patients according to the c-MET, RhoA, and CLDN18 groups are presented in Figure 3.

DFS was worse for c-MET-negative GC compared to c-MET-positive group (*p* = 0.008). Furthermore, low-RhoA patients had a significantly poor survival than patients with high-RhoA GC (*p* = 0.009). For CLDN18, DFS was similar between the low and high-CLDN18 groups (*p* = 0.719).

Regarding OS, there was no significant difference in survival between the groups for c-MET, RhoA, and CLDN18.

Univariate and multivariate analyses were performed to evaluate the prognostic factors affecting DFS and OS (Table 4). The DFS analysis revealed that the extent of gastrectomy, pT stage, and pN stage were factors to show independent prognostic significances. c-MET positive and high-RhoA demonstrated a significant association with worse survival in univariate analysis, whereas they did not exhibit statistical significance in the multivariate model. Regarding OS, multivariate analysis identified total gastrectomy, pT3/T4 status, and lymph node metastasis as independent factors associated with worse survival.

## 4. Discussion

Currently, several GC classifications have been proposed to improve the prognosis of patients by achieving individualized treatment and guiding clinical decision-making [4,25,26,27]. However, although prognostic significance and targets of therapeutic intervention of MSI and EBV subtypes are well established, the remaining subtypes comprise more heterogeneous groups of GC with varying characteristics and degrees of aggressiveness, resulting in different oncological outcomes [9,25]. Therefore, the identification of prognostic biomarkers to intervention is still necessary to understand the GC heterogeneity and improve patient survival. 

Accordingly, this study was conducted to investigate the protein expression of three alternative biomarkers related to the CIN and GS molecular subtypes in large and comprehensive cohort of patients with GC. c-MET, RhoA, and CLDN18 were examined by IHC, and evaluated according to the clinicopathological characteristics and survival. In addition, HER2 and E-cadherin expression―which are widely recognized by the association with subtypes CIN and GS, respectively [4,28]―were also assessed to better verify the relationship between markers and molecular subtypes.

Our findings demonstrated that the expression of c-MET, RhoA, and CLD18 occurs frequently in GC and that the relationship between them and other biomarkers, such as HER2 and E-cadherin, may support the association of these markers with the subtypes of the molecular classification. Furthermore, although they were not independent factors, RhoA and c-MET were associated with survival, and can be investigated as alternative biomarkers for patient stratification in subgroups of GC.

Currently, molecular parameters and predictors of prognoses in GC were investigated in various studies [29,30,31]. However, the immunoreactivity of c-MET, RhoA, and CLDN18 in GC regarding survival and clinicopathological aspects led to contradictory results. 

Similar to HER2, c-MET is another receptor tyrosine kinase overexpressed and activated in a subset of human epithelial malignancy. Aberrant c-MET pathway activation plays an important role in tumorigenesis as it promotes tumor cell growth, survival, migration, and invasion, as well as tumor angiogenesis [13]. In GC, it can occur mainly by protein overexpression, with frequencies of c-MET protein overexpression varying between 23% and 73% [32,33,34]. In our cohort, the c-MET staining pattern was positive in 48.4% of CG—similar to reported by Nakajima M et al. (46.1%) [34].

In previous studies, no significant correlation between c-MET expression and clinicopathological parameters were seen [33,35]. Conversely, some authors reported that c-MET overexpression was associated with depth of tumor invasion and lymph node metastasis [34]. It was reported that diffuse or mixed-type cancers were mostly c-MET-negative, and the score of IHC2+ was correlated with intestinal-type GC and less advanced TNM stages than those with IHC-negative. Moreover, only IHC3+ was associated with the advanced stage [32], which may justify the variation of results observed in the literature. In our study, we also observed an association between c-MET-negative and poorly differentiated histology. Although the frequency of lymph node metastasis and depth of tumor invasion had been higher in c-MET-negative cases, there was no significant association between c-MET and tumor stage. Nevertheless, we demonstrated the association between c-MET and HER2 protein expression, supporting the relationship of c-MET with tumors that have tyrosine kinase receptor hyperexpression—characteristic of the CIN subtype [4]. Interestingly, we also showed that c-MET was positively correlated with RhoA expression and negatively with CLDN18 expression—both related to the GS subtype [4].

Particularly, the GS subtype is associated with CDH1 and RhoA gene mutations, which morphologically corresponds to the diffuse Lauren type [4]. Furthermore, the TCGA study also reported the fusion between the CLDN18 and ARHGAP26 genes in this subtype, especially GC with RhoA mutation [4]. In our analysis, low-RhoA GC was associated with loss of E-cadherin expression, suggesting its relationship with the GS subtype.

RhoA is a GTPase protein of the Rho family which plays various key roles in biological processes, including cytoskeletal remodeling, cell migration, growth, and adhesion [11]. RhoA mutations or abnormal expression have been reported to be closely associated with the development of malignant tumors [11,36]. RhoA mutations lead to the loss of RhoA function, thus enabling the cells to gain the ability to resist an apoptotic process that acts as a barrier to metastasis known as “anoikis”—a form of cell death due to loss of contact with the extracellular matrix or neighboring cells—which promotes the infiltration and diffuse growth of cells [11,36]. Thus, it has also been correlated with poor survival and unfavorable prognostic in GC.

Studies evaluating RhoA expression in patients with GC show quite divergent results. Some authors showed a significantly higher expression of RhoA in diffuse GC, but no difference between RhoA IHC staining and TNM stage [37], while others demonstrated that advanced stages are associated with higher RHOA protein expression compared to early TNM stages [38]. In the present study, low-RhoA GC was associated with diffuse Lauren type, advanced pT, and TNM stage. Our results were consistent with previous analyzes that demonstrate a clinical association between high-RhoA expression with early-stage GC [15]. 

Meanwhile, CLDN18 is a component of tight junctions targeted as a differentiation molecule that is highly specific for the gastric mucosa [14,39]. In GC, 82.5% of our cases had some positivity for CLDN18 expression, which was similar to that found in other series (74.4%). Reduced or loss of its expression has been found to promote cell invasion and metastasis, leading to a poor prognosis [12]. In our study, there was no difference in CLDN18 expression between the Lauren types or even between the others clinicopathological characteristics. Although the cases with low-CLDN18 showed more advanced pT, this difference was not significant. Our result was similar to that reported by Dottermusch M. et al. [14], where no correlation was found between CLDN18 and Lauren phenotype, or among other clinicopathological characteristic [14].

Concerning survival outcomes, conflicting results have also been described as to whether these markers are a favorable or adverse prognostic factor in GC [12,14,20,21,32,40,41,42]. According to our study, a survival benefit in DFS was seen in patients who had c-MET-positive and high-RhoA, but the positivity itself for both did not confer a more favorable prognosis in multivariate analysis. This finding is consistent with previous observations. Regarding CLDN18, as well as in our cohort, its expression was not associated with survival outcomes [12,14].

Some methodological aspects that limit the interpretation of outcomes should be mentioned and can be partially responsible for the conflicting results. In the case of c-MET, for example, in a study performed by Lee HE et al. [32], high c-MET expression was associated with worse survival. However, they found that patients with a score of IHC2+ (*n* = 94) have better survival compared to a score of IHC0/1+(*n* = 334), and the worst survival is restricted only to those with a score of IHC3+, represented by only 10 patients where almost comprises stage IV GC [32]. For RhoA, the high expression of RhoA was generally associated with a worse prognosis in GC patients, while the mutation of the gene had no impact on survival [21,41,42]. However, initial studies on RhoA were based on cancer-associated mutations in Ras that supported an oncogene role for RhoA. More recently, sequencing studies have raised the possibility that RhoA may have a tumor suppression tumor function [43,44]. Our results, therefore, resemble more recent findings, where the low-RhoA expression appears to be associated with a poor prognosis [45]. The reduced expression of RhoA increased the risk of metastasis in breast cancer [45], and the loss of RhoA contributed to the current of metastasis in colon cancer [46]. Therefore, studies that evaluate RhoA mutation, not only for a gain-of-function mutation, and investigate the relationship with the RhoA protein expression are still needed.

Some limitations of this study should be mentioned. The present study is a retrospective analysis and there is a potential for selection bias since only patients undergoing gastrectomy were included. Therefore, we do not know the expression of these markers in the palliative setting. Furthermore, there is no standardized method to evaluate the IHC results for c-MET, RhoA, and CLDN18. Therefore, differences in results between studies may be due to variations in IHC grading schemes, antibody clones, scores systems, and cut-off values. 

Indeed, most studies were limited by small cohort sizes, and there were few studies conducted on a large and consecutive unselected cohort of GC patients. Some researchers are restricted only to tumors of the intestinal type, or only of the diffuse type [35,41,47], limiting the interpretation of the results. Furthermore, we analyzed c-MET and HER2 only for protein expression using IHC, and not for gene amplification.

Nevertheless, our study included a large and well-characterized cohort of GC patients (*n* = 349). To increase the reliability of the results, only curative patients were included, and results were achieved in a real-world setting. Based on their strong association with CIN and GS, we also evaluated HER2 and E-cadherin expression, which can provide additional information related to molecular subtypes. 

Currently, biomarker-based patient selection remains a challenge. In the last few years, c-MET, RhoA, and CLDN18 have been suggested as promising target options for GC therapy. Although Trastuzumab, which specifically targets HER2, is the recommended treatment for GC with HER2 overexpressing, only 20% of patients have an indication for therapy [48]. Thus, it was imperative to look for alternative options. In this sense, monoclonal antibodies against c-MET and HGF are being tested in ongoing clinical trials, suggesting that interruption of the HGF–MET axis has antitumor effects in GC cells [13]. Furthermore, it was shown that RhoA can be targeted by small molecule inhibitors, implicating it as a potential druggable target especially in diffuse GC [42]. Besides, clinical trials (FAST study) which investigated CLDN18 tumor expression and therapy with anti-CLDN18 demonstrated that Zolbetuximab (IMAB362) in combination with first-line chemotherapy provides a clinically relevant benefit in patients with inoperable or recurrent gastric and gastro-esophageal junction cancer [49,50]. Moreover, RhoA and CLDN18 represent potential biomarkers for GS, a poor-prognosis subtype of GC with no currently available molecularly targeted drugs.

## 5. Conclusions

The expressions of c-MET, RhoA, and CLD18 were frequently in GC. RhoA was related to distinct clinicopathological characteristics. Both c-MET-negative and low-RhoA were associated with unfavorable survival but were not independent predictors of poor prognosis. Thus, further investigations across different populations and subgroups of GC are still required to enhance the oncological outcomes and assist in patient stratification for treatments and prognostic groups.

## Figures and Tables

**Table 1:** Clinicopathological characteristics of gastric cancer patients according to the c-MET groups.

Variables		c-MET-Negative *n* = 180 (%)	c-MET-Positive *n* = 169 (%)	*p*
**Sex**				0.358
	Female	68 (37.8)	72 (42.6)	
	Male	112 (62.2)	97 (57.4)	
**Age (years)**				0.173
	Mean (SD)	63.1 (11.9)	61.4 (11.6)	
**ASA classification**				0.686
	I/II	155 (86.1)	148 (87.6)	
	III/IV	25 (13.9)	21 (12.4)	
**Type of resection**				**0.026**
	Subtotal	83 (46.1)	98 (58)	
	Total	97 (53.9)	71 (42)	
**Tumor size (cm)**				0.143
	Mean (SD)	5.3 (3.2)	4.8 (3.3)	
**Lauren type**				0.619
	Intestinal	90 (50)	89 (52.7)	
	Diffuse/mixed	90 (50)	80 (47.3)	
**Grade of histological differentiation**				**0.004**
	Well/moderately differentiated	68 (37.8)	90(53.3)	
	Poorly differentiated	112 (62.2)	79 (46.7)	
**Peritumoral inflammatory infiltrate**				**0.001**
	Absent/mild	104 (58.4)	128 (75.7)	
	Moderate/intense	74 (41.6)	41 (24.3)	
**Lymphatic invasion**				0.097
	No	82 (45.6)	92 (54.4)	
	Yes	98 (54.4)	77 (45.6)	
**Venous invasion**				0.680
	No	122 (67.8)	118 (69.8)	
	Yes	58 (32.2)	51 (30.2)	
**Perineural Invasion**				0.879
	No	88 (48.9)	84 (49.7)	
	Yes	91 (51.1)	85 (50.3)	
**pT status**				0.058
	pT1/T2	59 (32.8)	72 (42.6)	
	pT3/T4	121 (67.2)	97 (57.4)	
**No of lymph nodes**				0.600
	Mean (SD)	38.6 (18.4)	39.6 (18.6)	
**pN status**				0.088
	pN0	70 (38.9)	81 (47.9)	
	pN+	110 (61.1)	88 (52.1)	
**pTNM status**				0.164
	I/II	91 (50.6)	98 (58)	
	III/IV	89 (49.4)	71 (42)	
**RhoA**				**<0.001**
	Low-RhoA	116 (64.4)	58 (34.3)	
	High-RhoA	64 (35.6)	111 (65.7)	
**Claudin 18**				0.122
	Low-CLDN18	82 (45.6)	91 (53.8)	
	High-CLDN18	90 (54.4)	78 (46.2)	
HER2 *				**0.014**
	HER2 (0/+1)	151 (86.8)	119 (76.3)	
	HER2 (+2/+3)	23 (13.2)	37 (23.7)	
E-cadherin *				**0.013**
	Normal	150 (86.7)	146 (94.8)	
	Loss of expression	23 (13.3)	8 (5.2)	

SD, standard deviation; ASA, American Society of Anesthesiologists, *p*-values in bold are statistically significant. * some cases not available.

**Table 2:** Clinicopathological characteristics of gastric cancer patients according to the RhoA groups.

Variables		Low-RhoA *n* = 174 (%)	High-RhoA *n* = 169 (%)	*p*
**Sex**				0.793
	Female	71 (40.8)	69 (39.4)	
	Male	103 (59.2)	106 (60.6)	
**Age (years)**				0.260
	Mean (SD)	61.5 (12.8)	63.0 (10.7)	
**ASA classification**				0.198
	I/II	147 (84.5)	156 (89.1)	
	III/IV	27 (15.5)	19 (10.9)	
**Type of resection**				0.871
	Subtotal	91 (52.3)	90 (51.4)	
	Total	83 (47.7)	85 (48.6)	
**Tumor size (cm)**				0.170
	Mean (SD)	5.3 (3.3)	4.8 (3.3)	
**Lauren type**				**<0.001**
	Intestinal	72 (41.4)	107 (61.1)	
	Diffuse/mixed	102 (58.6)	68 (38.9)	
**Grade of histological differentiation**				
	Well/moderately differentiated	62 (35.6)	96 (54.9)	**<0.001**
	Poorly differentiated	112 (64.4)	79 (45.1)	
**Peritumoral inflammatory infiltrate**				**0.012**
	Absent/mild	104 (60.5)	128 (73.1)	
	Moderate/intense	68 (39.5)	47 (26.9)	
**Lymphatic invasion**				0.097
	No	79 (45.4)	95 (54.3)	
	Yes	95 (54.6)	80 (45.7)	
**Venous invasion**				0.191
	No	114 (65.5)	126 (72)	
	Yes	60 (34.5)	49 (28)	
**Perineural Invasion**				0.872
	No	85 (48.9)	87 (49.7)	
	Yes	89 (51.1)	88 (50.3)	
**pT status**				**0.023**
	pT1/T2	55 (31.6)	76 (43.4)	
	pT3/T4	119 (68.4)	99 (56.6)	
**No of lymph nodes**				0.269
	Mean (SD)	40.2 (18.1)	38.0 (18.8)	
**pN status**				0.355
	pN0	71 (40.8)	80 (45.7)	
	pN+	103 (59.2)	95 (54.3)	
**pTNM status**				**0.047**
	I/II	85 (48.9)	104 (59.4)	
	III/IV	89 (51.1)	71 (40.6)	
**c-MET**				**<0.001**
	c-MET-negative	116 (66.7)	64 (36.6)	
	c-MET-positive	58 (33.3)	111 (63.4)	
**Claudin 18**				0.422
	Low-CLDN18	90 (51.7)	83 (47.4)	
	High-CLDN18	84 (48.3)	91 (52.6)	
HER2 *				0.533
	HER2 (0/+1)	138 (83.1)	132 (80.5)	
	HER2 (+2/+3)	28 (16.9)	32 (19.5)	
E-cadherin *				**0.040**
	Normal	143 (87.2)	153 (93.9)	
	Loss of expression	21 (12.8)	10 (6.1)	

SD, standard deviation; ASA, American Society of Anesthesiologists. *p*-values in bold are statistically significant. * some cases not available.

**Table 3:** Clinicopathological characteristics of gastric cancer patients according to the CLDN18 groups.

Variables		Low-CLDN18 *n* = 173 (%)	High-CLDN18 *n* = 176 (%)	*p*
**Sex**				0.931
	Female	69 (39.3)	71 (40.3)	
	Male	104 (60.1)	105 (59.7)	
**Age (years)**				0.407
	Mean (SD)	62.8 (12.0)	61.7 (11.6)	
**ASA classification**				0.705
	I/II	149 (86.1)	154 (87.5)	
	III/IV	24 (13.9)	22 (12.5)	
**Type of resection**				0.258
	Subtotal	95 (54.9)	86 (48.9)	
	Total	78 (45.1)	90 (51.1)	
**Tumor size (cm)**				0.145
	Mean (SD)	5.3 (3.4)	4.8 (3.1)	
**Lauren type**				0.627
	Intestinal	91 (52.6)	88 (50)	
	Diffuse/mixed	82 (47.4)	88 (50)	
**Grade of histological differentiation**				0.151
	Well/moderately differentiated	85 (49.1)	73 (41.5)	
	Poorly differentiated	88 (50.9)	103 (58.5)	
**Peritumoral inflammatory infiltrate**				0.080
	Absent/mild	122 (71,3)	110 (62.5)	
	Moderate/intense	49 (28.7)	66 (37.5)	
**Lymphatic invasion**				0.120
	No	79 (45.7)	95 (54)	
	Yes	94 (54.3)	81 (46)	
**Venous invasion**				**0.038**
	No	110 (63.6)	130 (73.9)	
	Yes	63 (36.4)	46 (26.1)	
**Perineural Invasion**				0.874
	No	86 (49.7)	86 (48.9)	
	Yes	87 (50.3)	90 (51.1)	
**pT status**				0.079
	pT1/T2	57 (32.9)	74 (42)	
	pT3/T4	116 (67.1)	102 (58)	
**No of lymph nodes**				0.562
	Mean (SD)	39.7 (17.8)	38.5 (19.1)	
**pN status**				0.854
	pN0	74 (42.8)	77 (43.8)	
	pN+	99 (57.2)	99 (56.2)	
**pTNM status**				0.883
	I/II	93 (53.8)	96 (54.5)	
	III/IV	80 (46.2)	80 (45.5)	
**c-MET**				0.122
	c-MET-negative	82 (47.4)	98 (55.7)	
	c-MET-positive	91 (52.6)	78 (44.3)	
**RhoA**				0.422
	Low-RhoA	90 (52)	84 (47.7)	
	High-RhoA	83 (48)	92 (52.3)	
HER2 *				**0.046**
	HER2 (0/+1)	128 (77.6)	142 (86.1)	
	HER2 (+2/+3)	37 (22.4)	23 (13.9)	
E-cadherin *				0.608
	Normal	148 (91.4)	148 (89.7)	
	Loss of expression	14 (8.6)	17 (10.3)	

SD, standard deviation; ASA, American Society of Anesthesiologists. *p*-values in bold are statistically significant. * some cases not available.

**Table 4:** Univariate and multivariate analysis for disease-free survival (DFS) and overall survival (OS).

**Disease-Free Survival**	**Univariate Analysis**			**Multivariate Analysis**		
**Variables**	**HR**	**95% CI**	***p***	**HR**	**95% CI**	***p***
Male (vs female)	1.12	0.75–1.66	0.579	―	―	―
Age > 65 (vs <65 years)	0.84	0.57–1.24	0.384	―	―	―
Total Gastrectomy (vs. subtotal)	2.52	1.69–3.76	**<0.001**	2.05	1.37–3.09	**0.001**
Diffuse/mixed Lauren type (vs. others)	1.65	0.12–2.44	**0.011**	1.11	0.74–1.65	0.620
pT3/T4 status (vs. pT1/T2)	7.68	4.00–14.75	**<0.001**	3.83	1.92–7.64	**<0.001**
pN+ (vs. pN0)	5.92	3.42–10.24	**<0.001**	3.32	1.86–5.92	**<0.001**
c-MET-negative (vs. c-MET-positive)	1.70	1.14–2.52	**0.009**	1.26	0.84–1.90	0.258
Low-RhoA (vs. High-RhoA)	1.67	1.13–2.47	**0.010**	1.35	0.90–2.02	0.152
Low-CLDN18 (vs. High-CLDN18)	0.93	0.64–1.37	0.719	―	―	―
non-CMT (vs. CMT)	0.72	0.48–1.07	0.099	―	―	―
**Overall Survival**	**Univariate Analysis**			**Multivariate Analysis**		
**Variables**	**HR**	**95% CI**	***p***	**HR**	**95% CI**	***p***
Male (vs. female)	1.21	0.87–1.67	0.253	―	―	―
Age > 65 (vs <65 years)	1.22	0.89–1.67	0.218	―	―	―
Total Gastrectomy (vs. subtotal)	2.08	1.51–2.86	**<0.001**	1.87	1.35–2.58	**<0.001**
Diffuse/mixed Lauren type (vs. others)	1.25	0.91–1.71	0.162	―	―	―
pT3/T4 status (vs. pT1/T2)	3.07	2.08–4.54	**<0.001**	2.21	1.44–3.39	**<0.001**
pN+ (vs. pN0)	2.55	1.79–3.62	**<0.001**	1.74	1.18–2.56	**0.005**
c-MET-negative (vs. c-MET-positive)	1.26	0.92–1.73	0.149	―	―	―
Low-RhoA (vs. High-RhoA)	1.08	0.79–1.47	0.648	―	―	―
Low-CLDN18 (vs. High-CLDN18)	1.11	0.81–1.51	0.525	―	―	―
non-CMT (vs. CMT)	1.01	0.74–1.39	0.932	―	―	―

SD, standard deviation; CI, confidence interval; HR, hazard ratio; CMT, chemotherapy. *p*-values in bold are statistically significant.

## Data Availability

No additional data are available.

## Supplementary Materials

The following are available online at https://www.mdpi.com/article/10.3390/medsci10010004/s1. Table S1: Clinicopathological characteristics of gastric cancer patients according to the c-MET, RhoA and CLDN18 groups. Table S2: Pearson correlation test for IHC markers.
