# Supplementary material for: RhoA, Claudin 18, and c-MET in Gastric Cancer: Clinicopathological Characteristics and Prognostic Significance in Curative Resected Patients
Source: Med Sci (Basel). 2021 Dec 29;10(1):4. doi: 10.3390/medsci10010004 (PMC8788521; doi:10.3390/medsci10010004)
Supplement: Supplementary file 1 [file medsci-10-00004-s001.zip › medsci-1504245-supplementary.pdf]

Supplementary Table S1. Clinicopathological characteristics of gastric cancer patients according to the c-MET, RhoA and CLDN18 groups.

Variables	c-MET (–)	c-MET(+)	*p*	Low-RHOA	High-RHOA	*p*	Low-CLDN18	High-CLDN18	*p*
	n = 180 (%)	*n* = 169 (%)		*n* = 174 (%)	*n* = 169 (%)		*n* = 173 (%)	*n* = 176 (%)	
**BMI (Kg/cm²)**			0.844			0.455			0.101
Mean (SD)	24.2 (5.3)	24.1 (5.4)		24.3 (5.5)	23.9 (5.1)		23.6 (5.3)	24.6 (5.3)	
**Hemoglobin (g/dL)**			0.222			0.443			0.562
Mean (SD)	12.0 (2.3)	12.3 (2.3)		12.0 (2.3)	12.2 (2.2)		12.2 (2.2)	12.1 (2.3)	
**Albumin (g/dL)**			0.792			0.653			0.521
Mean (SD)	4.1 (2.4)	4.1 (0.6)		4.1 (2.5)	4.1 (0.5)		4.2 (2.5)	4.0 (0.6)	
**Neutrophil lymphocyte ratio (NLR)**			0.743			0.648			0.493
Mean (SD)	2.74 (3.00)	2.64 (2.58)		2.62 (2.89)	2.76 (2.76)		2.80 (3.01)	2.60 (5.56)	
**Charlson–Deyo Comorbidity Index (CCI)**			0.710			0.524			0.801
0–1	168 (93.3)	156 (92.3)		160 (92)	164 (93.7)		160 (92.5)	164 (93.2)	
> 1	12 (6.7)	12 (7.7)		14 (8)	11 (6.3)		13 (7.5)	12 (6.8)	
**pM status**			0.809			0.337			0.749
pM0	175 (97.2)	165 (97.6)		168 (96.6)	172 (98.3)		168 (97.1)	172 (97.7)	
pM1	5 (2.8)	4 (2.4)		6 (3.3)	3 (1.7)		5 (2.9)	4 (2.3)	
**c-MET (H-score)**			—			**<0.001**			**0.031**
Mean (SD)	—	—		12.7 (31.6)	28.6 (39.9)		24.9 (38.6)	16.5 (34.5)	
**RhoA (%)**			**<0.001**			—			0.185
Mean (SD)	26.2 (28)	46.9 (28.3)		—	—		34.1 (30.6)	38.3 (29.2)	
**Claudin 18 (%)**			0.113			0.989			0.185
Mean (SD)	37.5 (29.9)	32.4 (29.8)		35.1 (30.5)	35.0 (29.4)		34.1 (30.6)	38.3 (29.2)	
**Postoperative complication (POC)**			0.693			0.462			0.602
No POC/Clavien I-II	155 (86.1)	143 (84.6)		151 (86.8)	147 (84)		146 (84.4)	152 (86.4)	
Clavien III-V	25 (13.9)	26 (15.4)		23 (13.2)	28 (16)		27 (15.6)	24 (13.6)	
**Chemotherapy (all)**			0.382			0.119			0.231
No	80 (44.4)	83 (49.1)		74 (42.5)	89 (50.9)		75 (43.4)	88 (50)	
Yes	100 (55.6)	86 (50.9)		100 (57.5)	86 (49.1)		98 (56.6)	88 (50)	
**Disease recurrence**			na			na			na
No	114 (63.3)	130 (79.9)		110 (63.2)	134 (76.6)		123 (71.1)	121 (68.8)	
Yes	66 (36.7)	39 (23.1)		64 (36.8)	41 (23.4)		50 (28.9)	55 (31.2)	
**Death**			na			na			na
No	91 (50.6)	101 (59.8)		94 (54)	98 (56)		93 (53.8)	99 (56.2)	
Yes	89 (49.4)	68 (40.2)		80 (46)	77 (44)		80 (46.2)	77 (43.8)	

BMI, body mass index; SD, standard deviation. *p*-values in bold are statistically significant.

Supplementary Table S2. Pearson correlation test for IHC markers

		**c-MET**	**RhoA**	**CLDN18**
**c-MET**	Pearson Correlation coefficient (r)	—	0.250	– 0.139
	p-value	—	<0.001	0.010
**RhoA**	Pearson Correlation coefficient (r)	0.250	—	0.023
	p-value	<0.001	—	0.664
**CLDN18**	Pearson Correlation coefficient (r)	– 0.139	0.023	—
	p-value	0.010	0.664	—
**HER2**	Pearson Correlation coefficient (r)	0.160	0.049	– 0.107
	p-value	0.002	0.376	0.052
**E-cadherin**	Pearson Correlation coefficient (r)	– 0.122	– 0.125	0.025
	p-value	0.028	0.024	0.657
